# Education and development of rehabilitation therapy in China under the background of aging

**DOI:** 10.3389/fpubh.2022.1000048

**Published:** 2022-12-07

**Authors:** Shuyue Sun, Xingru Lin, Xiaoyao Ouyang, Weiqin Cai, Qianqian Gao, Peiwu Guo, Zongrun Li, Lihong Ji, Zhiwei Dong, Qi Jing, Jianhua Zhang

**Affiliations:** ^1^School of Management, Weifang Medical University, Weifang, China; ^2^School of Management, China Rehabilitation and Health Institute, Weifang Medical University, Weifang, China; ^3^Healthy Shandong Risk Prediction and Early Warning Collaborative Center, Weifang Medical University, Weifang, China; ^4^School of Public Health, Weifang Medical University, Weifang, China; ^5^Rehabilitation Department, The First Affiliated Hospital of Weifang Medical University/Weifang People's Hospital, Weifang, China; ^6^School of Rehabilitation Medicine, Weifang Medical University, Weifang, China

**Keywords:** aging, talent development, China, education, rehabilitation

## Abstract

The World Health Organization (WHO) estimates that about one-third of people worldwide currently have a need for rehabilitation. This demand is expected to increase in the coming years due to changes in population health and characteristics. For example, with the intensification of global aging, the rehabilitation needs of patients with chronic diseases and disabilities, postoperative dysfunction, and cognitive impairment continue to grow, and emergencies such as conflicts, disasters, and epidemics may lead to a surge in rehabilitation needs. Early and sustained rehabilitation could reduce complications, improve function, and reduce disability in affected populations, but rehabilitation services are often underestimated due to underfunding and poor short-term outcomes. WHO sees rehabilitation as an essential part of achieving universal health coverage and the Sustainable Development Goals. With the development of China's economy and society and the trend of an aging population, the demand for rehabilitation therapists is growing rapidly. Since the development of rehabilitation education in China at the beginning of this century, great progress has been made in both the training mode and the number of people trained, especially in the construction of higher education of rehabilitation in related colleges and universities. Through descriptive qualitative analysis, this study collected information from national policy documents and official websites of colleges and universities on policies concerning degree systems, cultivating goals and ideas, courses, education internationalization, continuing education in practice, standardized training after graduation, the number of colleges and universities with rehabilitation therapy related majors in China, and summarizes the current situation of the development of rehabilitation personnel education in Chinese colleges and universities. Judging from the results, during the development of rehabilitation education, China has continuously improved in terms of policy support, educational goals and concepts, the number and quality of institutions, degree systems, and internationalization, while gradually adapting to China's development status. This also provides direction and feasible suggestions for China to improve the rehabilitation education development system and formulate a national rehabilitation education plan in the future to deal with the challenge of aging.

## Introduction

With the increase in global life expectancy and changes in the health status of populations caused by disabilities, chronic diseases, postoperative dysfunction, cognitive impairments, etc., the demand for professional rehabilitation therapists in various countries has increased significantly. But in many countries, especially low- and middle-income countries, this growing demand for rehabilitation is largely unmet, with an average of fewer than 10 skilled practitioners per 1 million people (1). In this context, how to improve the quantity and quality of rehabilitation therapists has become a key issue in the development of rehabilitation therapy education.

Currently, many countries in the world have gradually formed a relatively complete rehabilitation therapy education system, according to the subdivision of rehabilitation therapy professional hierarchical training, talent training channels and methods are more diversified, and advanced concepts such as ICF are integrated into the educational concept so that the training of rehabilitation therapy talents is more in line with the current actual needs. Rehabilitation education originated in the 1950s, and gradually diverged into physiotherapists, occupational therapists, and speech therapists in the 1970s. Currently, most of the international rehabilitation education system is 4 year university education and postgraduate education, awarded bachelor of science, master's, and doctoral degrees, and in a few countries, still 3 years of specialist education exists. In accordance with the requirements of the World Confederation for Physical Therapists (WCPT) and the World Federation of Occupational Therapists (WFOT), many institutions have established a comprehensive high-level normative training system, including education, scientific research, and further education. The qualification examination is carried out according to the professional scope of the rehabilitation therapist. The employment of graduates of rehabilitation therapy-related majors is mainly for the rehabilitation departments, community rehabilitation institutions, nursing homes, fitness centers, sports clubs, and sports training equipment management institutions of hospitals at all levels, and there are also individual operators (2, 3). By discussing and comparing the development status of rehabilitation education in the United States, the United Kingdom, Japan, and other countries, it can be seen that the development of rehabilitation education in various countries has a common trend.

To meet the rehabilitation needs of the population in the context of today's aging and population health, countries have taken various measures to improve the level of rehabilitation in their countries, with special attention to improving rehabilitation education, which is the fundamental way to develop rehabilitation therapists. For example, according to the needs of national social and economic development, the training of bachelor of rehabilitation in Japan highlights the acceptance of clinical internships in stages, and the internship institutions are not limited to medical rehabilitation institutions, but also include health and welfare institutions to enhance the comprehensive competence of talents in an all-round way (4). The Australian and New Zealand Physiotherapy Practice Thresholds in Australia and Aotearoa New Zealand (5) jointly developed by the Australian and New Zealand Physiotherapy Councils in 2015, which clearly describes the basic level of competency required for a registered physiotherapist to practice includes: knowledge, skills, attitudes, values, and judgment to effectively and comprehensively measure the competence of a physiotherapist (6).

As a large country with a population of more than 1.4 billion and accounting for nearly one-third of the world (7), China faces multiple challenges such as a high degree of population aging and rapid growth, a high incidence of chronic diseases, and an increase in the number of people with disabilities and postoperative rehabilitation, and the demand for rehabilitation therapy is relatively large. At the end of 2019, China's population aged 60 and over was close to 254 million, accounting for 18.1% of the world's elderly population (8). As of November 2020, China's population aged 60 and over has exceeded 264 million, accounting for 18.70% of the country's total population, an increase of 5.44 percentage points compared with 2010 (9). Therefore, the level of rehabilitation therapist development is crucial to global health promotion. China has also done a lot of work in this regard in recent years and achieved good results. Since about the 1990s, some colleges and universities in China have offered rehabilitation-related majors (10). In 2001, the Ministry of Education officially approved five colleges and universities, including Capital Medical University, to open undergraduate education in rehabilitation therapy, with a 4 year academic system and a bachelor of science degree (11), thus opening up the cultivation of rehabilitation therapists in China (12). After more than 20 years of development, China's professional and technical personnel engaged in rehabilitation therapy have exceeded 14,000 people (13), more than 100 colleges and universities have established undergraduate education in rehabilitation therapy, nearly 500 colleges and universities have set up rehabilitation therapy related professional disciplines, and initially established a multi-level rehabilitation therapy talent training system of specialty (vocational)–undergraduate–master–doctoral degree and the world's first University of Health and Rehabilitation Sciences, which has made a top-level design for the comprehensive development and refinement of rehabilitation therapy education. In terms of integrating with the international rehabilitation therapy concept and education model, Capital Medical University, Kunming Medical University, Sun Yat-sen University, Sichuan University, and other universities are at the forefront, and they refer to the international curriculum system and combine the actual development of rehabilitation therapy in China and integrate into the development advantages of the institutions themselves, contributing a better Chinese model to the world's rehabilitation therapy higher education.

This study aims to sort out the development status of mainland China rehabilitation therapy-related majors in the four education levels of professional(vocational)–undergraduate–master's and doctoral degrees, including the development of colleges and universities, educational objectives, professional categories, etc. at each education level, which is of great significance for relevant scholars and public health policymakers to improve the high-level education concept of rehabilitation therapy in the field of aging health and healthcare, and to establish a rehabilitation therapy education development plan that is more in line with China's actual situation.

## Materials and methods

### Data sources

The data sources of this study mainly include (1) Policy documents: Search for policy documents related to rehabilitation education, training, and career development on the official websites of the Ministry of Education of the People's Republic of China and the National Health Commission of the People's Republic of China. (2) Public information of relevant colleges and international organizations: The public information from the China Graduate Admissions Information Network, the World Physiotherapy Alliance, the World Occupational Therapy Alliance, and some colleges and universities in China has been collated, including rehabilitation therapy related professional training programs and curriculum settings, the rehabilitation therapy education of relevant colleges and universities meets international standards, and published literature: search for literature related to rehabilitation and education on CNKI, Wanfang Data Knowledge Service Platform, VIP, PubMed, Web of Science, and other domestic and foreign websites.

### Method

This study used descriptive qualitative analysis, which mainly contained information on relevant colleges and majors in each training level of rehabilitation education in China. First of all, the educational objectives of rehabilitation therapy-related majors at different training levels were listed and their characteristics were summarized and analyzed, and then the number of institutions involved in each training level was counted, and finally, the level and category of relevant institutions, the regions they belong to, the types of degrees they open, the time of opening and the training years, etc. were analyzed to further analyze the development ideas, deficiencies, and countermeasures of China's rehabilitation education to lay the foundation.

## Results

### Relevant policies support is gradually improved

In 2013, the “Several Opinions of the State Council on Accelerating the Development of the Old-age Service Industry” and the “Several Opinions of the State Council on Promoting the Development of the Health Service Industry” proposed to scientifically set up professional and technical posts in old-age institutions, focus on training and introducing rehabilitation therapists and other professional and technical personnel with professional or vocational qualifications, increase the training and vocational training of health service personnel, and standardize and accelerate the training of practitioners including rehabilitation therapists. In 2015, the Guiding Opinions of the General Office of the State Council on Promoting the Construction of a Graded Diagnosis and therapy System once again emphasized the need to strengthen the training of professionals such as rehabilitation therapists in various ways, strengthen the construction of grass-roots medical and health personnel, and meet the multi-level and diversified health service needs of the people. In 2016, the “Several Opinions of the State Council on Accelerating the Development of the Rehabilitation Assistive Devices Industry” listed the teamwork of rehabilitation physicians, rehabilitation therapists, and rehabilitation assistive device configuration personnel as the focus of development. In 2017, to further meet the rehabilitation needs of the elderly, China focused on strengthening the training of rehabilitation therapists, rehabilitation physicians, and rehabilitation assistive devices in the “Notice of the State Council on Printing and Distributing the 13th Five-Year Plan for the Development of the National Aging Cause and the Construction of the Pension System,” and extensively carried out various types of rehabilitation training services for the elderly.

### Educational goals and ideas are gradually clarified

Modern rehabilitation has been introduced to China in the 1980s and has only developed for a few decades. According to literature and relevant statistics, many Chinese colleges and universities have been carrying out research and education degree training in the field of rehabilitation therapy since the end of the 1980s. In 2004, the Rehabilitation Medicine Education Professional Committee of the Chinese Rehabilitation Medicine Association put forward the “Rehabilitation therapy Professional and Technical Personnel Access Standards” and “Undergraduate Rehabilitation Therapy Professional Education Setting Standards,” which clearly defined the concept of rehabilitation therapists, and made clear requirements for the professional skills they mastered. The personnel training targets of rehabilitation and therapy-related majors in Chinese educational levels are listed below (Table 1).

**Table 1:** Educational objectives of each training level of rehabilitation.

**Cultivating category**	**College (Example)**	**Major (Example)**	**Educational objectives**
Junior college	Guangzhou Health Science College	Rehabilitation technology	Master the professional knowledge and technical skills of rehabilitation technology, oriented to the position group of rehabilitation therapists in health and social work and other industries, capable of physical therapy, occupational therapy, speech therapy and other work of high-quality technical skills.
Undergraduate	Weifang Medical University	Rehabilitation techniques	Training to meet the need of the development of Chinese medical and health undertakings, has the good professional quality and humanistic accomplishment, with basic medicine, clinical medicine, traditional Chinese medicine base knowledge, a solid system of rehabilitation medicine basic theory and the stronger rehabilitation therapy technology, immunity-lower disease rehabilitation therapy and evaluation work ability, life-long learning ability and innovation consciousness, Applied technical personnel capable of rehabilitation evaluation and therapy in medical and health units and rehabilitation institutions.
Master	Zhejiang University	Sports medicine (sports rehabilitation)	Master comprehensive and solid basic theories and professional knowledge of sports medicine, understand the frontier and trends of sports medicine discipline development, have the ability to engage in sports rehabilitation, sports health care, sports training monitoring and other aspects of the research work, or independently undertake the professional technical work. Combine theory with practice, have innovative consciousness, can analyze and solve the new issues emerging in the development of modern medical science and interdisciplinary, and independently carry out research work in sports medicine discipline and related disciplines.
Doctor	Fudan University	Rehabilitation medicine and physiotherapy	With solid basic theoretical knowledge of clinical medicine and knowledge of rehabilitation medicine, deeply understand the latest research results and developments of rehabilitation medicine, master the diagnosis and therapy technology of rehabilitation medicine, and can independently undertake the diagnosis and therapy, scientific research and teaching of rehabilitation medicine.

Rehabilitation education at the specialist (vocational college) level emphasizes the mastery and proficiency of relevant skills. Currently, Chinese colleges and universities are gradually paying attention to physical therapy and sports therapy, while they are relatively weak in occupational therapy and speech therapy, and they also lack attention to traditional Chinese rehabilitation skills, such as TCM rehabilitation and Tuina. The undergraduate degree in rehabilitation therapy focuses more on translating theoretical knowledge into practical experience and cultivating application-oriented professionals who can engage in rehabilitation evaluation and rehabilitation therapy in medical and health units and rehabilitation institutions. Compared with the specialty, the undergraduate program has higher requirements for students' lifelong learning ability and sense of innovation. Postgraduate education requires students to go hand in hand with theoretical learning, operational exercises, and subject research so that they can continuously discover scientific problems in clinical practice and carry out creative scientific inquiries and scientific research activities.

In addition, graduate education in rehabilitation can provide a clearer career framework for therapists. Obviously, vigorously developing postgraduate education in rehabilitation therapy is an important way to improve the overall level of rehabilitation therapy, and it is also the general trend in the development of rehabilitation education in China.

### The number of colleges and universities with rehabilitation related majors has increased year by year

**Figure 1:**
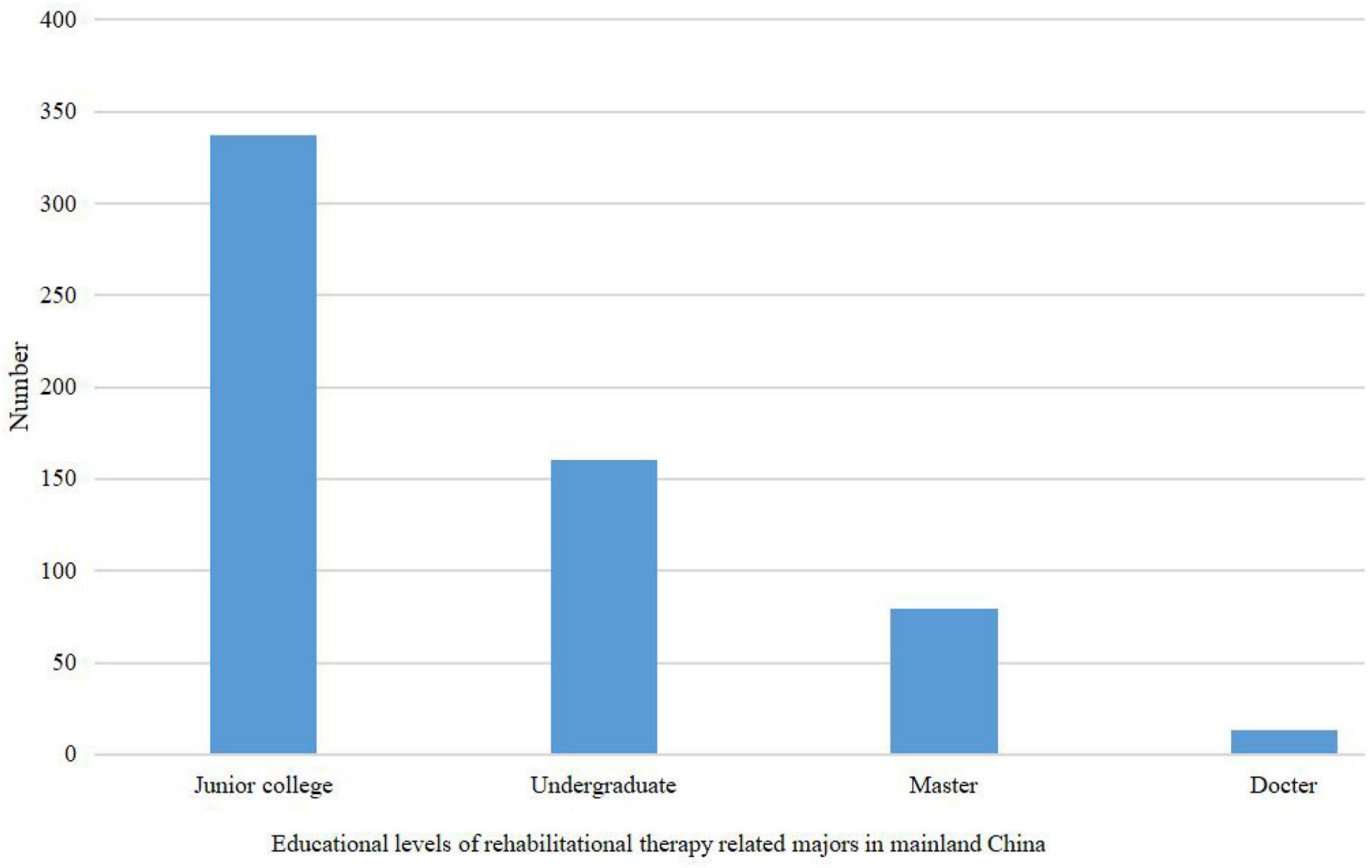
Number of colleges and universities at various levels of education in rehabilitation therapy-related majors in China (according to incomplete statistics by authors).

In recent years, the number of colleges and universities officially approved by the Ministry of Education to open rehabilitation therapy majors has increased year by year, especially high-level colleges. As of 2022, the statistics of various education levels of rehabilitation therapy related majors in China are carried out, and the results are as follows (Figure 1); from the figure, it can be seen that the educational levels of China's rehabilitation therapy related majors are still mainly specialized (vocational) and undergraduate education, and the development of master's and doctoral degrees is relatively weak. According to the collection of professional information related to rehabilitation therapy in various colleges and universities, a total of 79 colleges and universities in mainland China carry out master's education in rehabilitation therapy related majors, and the provinces (municipalities directly under the central government) with 5 or more master's programs are Beijing (8), Shanghai (6), Liaoning (6), Shandong (5), and Sichuan (5), all of which are provinces with more developed medical and health industries. Thirteen colleges and universities carry out doctoral education in rehabilitation related majors, of which seven are comprehensive colleges and universities with a level of 985,211 and double first-class, and the remaining six are provincial medical and health colleges. For information on colleges and universities related to rehabilitation therapy in mainland China at all levels of education (Table 2).

**Table 2:** Rehabilitation-related professional colleges and universities at all levels of education in China.

**Cultivating category**	**Major**	**College/University**	**Degree**	**College level**	**College/University classes**	**Region**	**Opening time**	**Cultivation of a fixed number of years**
Junior college	Rehabilitation technology	Guangzhou Health Science College		Higher vocational college	Medical and health	Guangdong	2003	3 years
		Yingkou Vocational and Technical College		Higher vocational college	Comprehensive	Liaoning	2019	3 years
	Speech and hearing rehabilitation technology	Changsha Social Work College		Higher vocational college	Comprehensive	Hunan	2014	3 years
		NingBo College of Health Sciences		Higher vocational college	Medical and health	Zhejiang	2014	3 years
	Traditional Chinese medicine rehabilitation technology	Jiangsu Health Vocational College		Higher vocational college	Medical and health	Jiangsu	2017	3 years
		Heze Medical College		Technical college	Medical and health	Shandong	2016	3 years
Undergraduate	Rehabilitation therapeutics	Harbin Medical University	Bachelor of Science	Provincial institutions of higher learning	Medical and health	Heilongjiang	2005	4 years
		China Medical University	Bachelor of Science	Provincial institutions of higher learning	Medical and health	Liaoning	2013	4 years
	Hearing and speech rehabilitation	Kunming Medical University	Bachelor of Science	Provincial institutions of higher learning	Medical and health	Yunnan	2014	4 years
		Shandong University of Traditional Chinese Medicine	Bachelor of Science	Provincial institutions of higher learning	Medical and health	Shandong	2017	4 years
	Rehabilitation therapy (physical therapy)	Capital Medical University	Bachelor of Science	Provincial institutions of higher learning	Medical and health	Beijing	2021	4 years
		Weifang Medical University	Bachelor of Science	Provincial institutions of higher learning	Medical and health	Shandong	2022	4 years
	Rehabilitation therapy (occupational therapy)	Capital Medical University	Bachelor of Science	Provincial institutions of higher learning	Medical and health	Beijing	2021	4 years
		Weifang Medical University	Bachelor of Science	Provincial institutions of higher learning	Medical and health	Shandong	2021	4 years
	Rehabilitation of traditional Chinese medicine	Heilongjiang University Of Chinese Medicine	Bachelor of Medicine	Provincial institutions of higher learning	Medical and health	Heilongjiang	2017	5 years
		Nanjing University Of Chinese Medicine	Bachelor of Medicine	Double First- Class	Medical and health	Jiangsu	2018	5 years
	Educational rehabilitation	East China Normal University	Bachelor of Education	Project 985, Project 211, Double First- Class	Normal	Shanghai	2015	4 years
		Nanjing Normal University of Special Education	Bachelor of Education	Regular undergraduate universities	Normal	Zhejiang	2017	4 years
Master	Rehabilitation medicine and physiotherapy	TianJin University of Sport	Master of Medicine	Provincial institutions of higher learning	Sports	Tianjin	2004	3 years
		Wenzhou Medical University	Master of Medicine	Provincial institutions of higher learning	Medical and health	Zhejiang	2006	3 years
	Sports medicine (sports rehabilitation)	Beijing Sport University	Master of Medicine	Project 211, Double First- Class	Sports	Beijing	2015	3 years
		Zhejiang University	Master of Medicine	Project 985, Project 211, Double First- Class	Comprehensive	Zhejiang	2021	3 years
	Sports rehabilitation	Wuhan Institute of Physical Education	Master of Science	Provincial institutions of higher learning	Sports	Hubei	2015	3 years
		Shandong Sport University	Master of Science	Provincial institutions of higher learning	Sports	Shandong	2015	3 years
	Medical technology (rehabilitation therapy)	China Medical University	Master of Medicine/Science	Provincial institutions of higher learning	Medical and health	Liaoning	2013	3 years
		Kunming Medical University	Master of Medicine/Science	Provincial institutions of higher learning	Medical and health	Yunnan	2018	3 years
Doctor	Rehabilitation medicine and physiotherapy	Fudan University	Doctor of Medicine	Project 985, Project 211, Double First- Class	Comprehensive	Shanghai	2005	4 years
		Xi'an Jiaotong University	Doctor of Medicine	Project 985, Project 211, Double First- Class	Comprehensive	Shaanxi	2017	4 years
	Medical technology	Sun Yat-sen University	Doctor of Medicine/Science	Project 985, Project 211, Double First- Class	Comprehensive	Guangdong	2018	4 years
		Sichuan University	Doctor of Medicine/Science	Project 985, Project 211, Double First- Class	Comprehensive	Sichuan	2018	3 years

From Table 2, it can be seen that the level of rehabilitation education in China mainly stays at the professional (vocational) level, the number of master's and doctoral colleges and universities is far lower than that of the professional (vocational) level, and the overall level of talent training needs to be improved. International rehabilitation therapy majors are mainly offered in some medical schools, science and engineering colleges, and scientific research institutes. For example, the University of Cambridge, New York University, the University of Sydney, and the Hong Kong Polytechnic University in Australia. Among them, more than 200 universities and research institutions in the United States carry out rehabilitation therapeutic education and research work (14), mainly to cultivate senior talents, and the above only cultivate or mainly train master of science and doctorate.

### There are slight differences between the degree systems of rehabilitation education in China and other countries

Most of the undergraduate education in rehabilitation therapy majors in mainland China is of 4 years, with a bachelor's degree in science, the 1st to 3rd year for theoretical courses, and the 4th academic year for the internship stage. After entering the clinic in the third academic year, most of them are divided into physical therapy (PT) and occupational therapy (OT) directions, and a few colleges and universities have also carried out speech therapy (ST) direction classes, focusing on strengthening the teaching of professional courses and clinical practice in their directions (15). There are not a few universities that have determined the establishment of master's programs, including Nanjing Medical University, Weifang Medical University, Shanghai University of Traditional Chinese Medicine, etc., mainly concentrated in the comprehensive colleges and universities, medical and health and sports colleges that have carried out postgraduate education in rehabilitation medicine and physiotherapy and undergraduate education in rehabilitation therapy earlier. Applicants must be rehabilitation therapy majors, sports rehabilitation majors, clinical medicine majors, etc., and have obtained medical and science degrees, full-time master's degrees are mostly 3 years of study, except for rehabilitation medicine and physiotherapy, sports medicine to obtain a master's degree in medicine, the rest of the master's degree is a master's degree in science.

Most period of rehabilitation therapy education is 4 years of undergraduate education, while postgraduate education is different. In the United States, the physical therapy major is a degree in education, but since 2014, the United States has phased out the master's degree in physical therapy, so the degree level is Doctor of Physical Therapy (DPT). Most students go through 4 years of undergraduate basic course study and clinical internship, and a few colleges and universities allow students who have completed 3 years of rehabilitation preparatory education to apply for 3 years of DPT study from higher institutions, the most common undergraduate majors are sports medicine, biomedicine, and sports science. In Japan, undergraduate rehabilitation therapy departments, such as Kyoto University and Capital University, mostly implement 4 year theoretical courses and annual internship courses, such as physical therapy and occupational therapy, and their master's and doctoral programs are implemented according to the credit system.

### The degree of rehabilitation education internationalization has been improved

Mainland China's rehabilitation education has gradually been in line with international standards in recent years, and the independent training of PT and OT with reference to WCPT and WFOT education standards is becoming the future trend of related professional education in the field of rehabilitation therapy in China. As the highest level and most authoritative organization in the field of PT and OT rehabilitation therapy in the world, WCPT and WFOT have promulgated the minimum education standards for physical/occupational therapists in the world to promote the development of physical/occupational therapists, and have absorbed the registration of many countries and their related colleges and universities. Since 2013, the PT and OT professional courses at Sichuan University, Shanghai University of Traditional Chinese Medicine, Capital Medical University, Kunming Medical University, Fujian University of Traditional Chinese Medicine, Tongji University, Guangzhou Medical University, Nanjing Medical University, and other eight institutions in China have successively passed the certification of WCPT and WFOT (16, 17) (Table 3), reflecting China's improvement in the internationalization of physical/occupational therapy education, which is also China's compliance with international standards. Proof of active exploration on the road of rehabilitation therapy professional construction with national characteristics.

**Table 3:** Colleges and universities certified by the WCPT/WFOT in mainland China.

**College/University**	**Physical therapy programme**	**Status awarded**	**Accreditation ends**	**Occupational therapy programme**	**Status awarded**	**Accreditation ends**
Capital Medical University	Bachelor Degree in Physical Therapy	2017	2022	Bachelor Degree of Occupational Therapy	2020	2024
Fujian University of Traditional Chinese Medicine	Bachelor Degree in Physical Therapy	2017	2022	Bachelor of Science Degree in Occupational Therapy	2020	2024
Kunming Medical University	Bachelor of Science in Physical Therapy	2017	2022	BSc Occupational Therapy	2020	2024
Shanghai University of Traditional Chinese Medicine	Bachelor of Science (BSc) in Rehabilitation Therapy (Physical Therapy)	2016	2023	Bachelor of Occupational Therapy	2019	2023
Sichuan University	Bachelor of Science in Physical Therapy	2017	2022	Bachelor of Occupational Therapy	2013	2027
Tongji University	Bachelor Degree in Physical Therapy	2017	2024			
Guangzhou Medical University				Bachelor Degree of Occupational Therapy	2020	2027
Nanjing Medical University				Bachelor of Science Degree in Occupational Therapy	2020	2027

In recent years, China's emphasis on rehabilitation-related majors has also been reflected in the construction of rehabilitation professional colleges. In 2015, the China Disabled Persons' Federation proposed to build the world's first University of Health and Rehabilitation Sciences and positioned it as a high-level national applied research university with international characteristics based on research and application-oriented with Chinese characteristics. Since then, the construction of the University of Health and Rehabilitation Sciences has been included in the “Outline of the 13th Five-Year Plan for National Economic and Social Development of the People's Republic of China” and the “13th Five-Year Plan for the Development of National Education.” In 2019, the Ministry of Education officially approved the preparation and support for the establishment of the University of Health and Rehabilitation Sciences, sponsored by Shandong Province, and jointly built by the China Disabled Persons' Federation, the National Health Commission, and other departments. On June 11, 2019, the University of Health and Rehabilitation Sciences held an unveiling ceremony in Qingdao, Shandong Province, and the Boao Forum for Asia Global Health Forum Conference was held in Qingdao on the same day. As one of the sub-forums, the “Seminar on rebuilding a life for the disabled—Building the University of Health and Rehabilitation Sciences” invited experts from the World Health Organization, Japan, New Zealand, the United Kingdom, Canada, Sweden, Finland, Hong Kong, China, and relevant universities and research institutions in China to participate, and put forward valuable suggestions on the educational philosophy and discipline construction of the University of Health and Rehabilitation Sciences.

### Rehabilitation therapy courses meet the health needs of the population

Proper curriculum arrangement is the basis for building knowledge and ability. Many colleges and universities related to rehabilitation in mainland China have carried out more refined courses, basically meeting the health needs of the population. For example, the School of Rehabilitation Medicine of Weifang Medical University has set up geriatric, community rehabilitation, and other courses (Table 4) concerning the elderly as the main research object. The content has almost covered information about cognitive impairment of the elderly, frailty, immobility, instability, urinary and fecal incontinence, communicative incapacity, isolation social, cognitive disability, and postural instability, which can meet the health needs of the population.

**Table 4:** Curriculum design based on Weifang Medical College.

**Professional compulsory courses**	**Professional elective courses**	**Senior related professional courses**
Communication skills between doctors and patients	Medical ethics	Gerontology
Human anatomy	Medical Physics	Community rehabilitation
Diagnostics	Physiology	
Neurology	Biochemistry	
Medicine	Pathology	
Osteology	Immunology	
Traditional rehabilitation methods of traditional Chinese medicine	Medical Statistics	
Introduction to Rehabilitation Therapy	Evidence-based medicine	
Rehabilitation Assessment	Science of health maintenance of traditional Chinese medicine	
Physiotherapy	Human Developments	
Occupational Therapy	Gerontology	
Clinical Rehabilitation Engineering	Pediatrics	
Speech Therapy	Introduction to Traditional Chinese Medicine	
Muscle and bone rehabilitation		
Rehabilitation of Neurology		
Rehabilitation of internal and external diseases		
Rehabilitation Psychology		
Community rehabilitation		

It is concluded that in the education of rehabilitation related majors in China, many colleges and universities have strengthened the teaching of geriatrics, community rehabilitation, and other courses, and paid attention to the training and education related to elderly rehabilitation in practical training. In addition, many colleges and universities' enterprise cooperation between institutions and rehabilitation related institutions with a high proportion of elderly people has been strengthened, such as community rehabilitation centers and geriatric rehabilitation departments in general hospitals, which has laid a foundation for promoting students' practical courses (18).

### Continuing education in practice is carried in many types

According to the research, the training of clinical ability of Chinese students majoring in rehabilitation therapy after graduation mainly depends on the clinical teaching level of medical institutions participating in an internship before graduation (19) and continuing education.

There are four main types of continuing education in practice that rehabilitation therapists participate in short-term continuing education project training, including national, provincial, and municipal short-term education projects, regular refresher education, 3 to 6 months of further learning in a superior hospital, participate in academic conferences and get continuing education credits, and online course learning. The above types of continuing education in practice have greatly promoted the profession of rehabilitation therapy. Taking Weifang People's Hospital as an example, the hospital strongly supported the continuing education in the practice of its rehabilitation therapists and provided sufficient funds and platform resources. The rehabilitation therapists of the hospital regularly go to the superior hospital for further study every year and regularly participate in training classes for various special rehabilitation therapists, each time for about 3 months or half a year, mainly for the training, teaching, exchange, and discussion of theory, operation technology, and actual cases. In addition, regular learning seminars are held within the department, and internal staff is organized to summarize and discuss recent cases to learn from experience. Finally, all employees will also take part in the online courses organized by the hospital. Online courses are not aimed at all sub-professional rehabilitation therapists but mainly focus on the comprehensive quality and medical ethics of staff. The credit system is usually implemented in online course learning, and the course can be concluded only after passing the corresponding online examination.

### Expert consensus on standardized training after graduation was proposed

Standardized training after graduation is an important means to further promote the comprehensive quality of Chinese rehabilitation therapists and improve their post-abilities, which has become the consensus of many experts in China (20). In recent decades, some medical schools in China, together with their affiliated hospitals, have carried out standardized training for rehabilitation therapists after graduation, most of which are 1 or 2 year courses. Colleges and universities that have standardized training for 1 year rehabilitation therapists after graduation, such as the First Affiliated Hospital of the University of Science and Technology of China and the First Affiliated Hospital of Nanchang University, have better improved the theoretical level of professional knowledge and post-ability of trainees through this training so that they can better meet the needs of clinical work. Some colleges and universities that carry out standardized training for 2 year rehabilitation therapists after graduation have more detailed and comprehensive training. For example, West China Hospital of Sichuan University and the Fifth Affiliated Hospital of Zhengzhou University not only conducted differentiated training on physical therapy and occupational therapy for trainees but also focused on sub-professional training on specialized rehabilitation, such as nerves, musculoskeletal, and cardiopulmonary. At the same time, with post-competency as the core, we focus on training students' clinical teaching ability, medical ethics, management ability, and other comprehensive qualities.

## Discussion

The development of rehabilitation staff is a powerful measure to cope with aging and chronic diseases, disabilities, and postoperative rehabilitation. Currently, the problem of population aging cannot be ignored, in addition to various chronic diseases and physiological decline, etc. are increasing the demand for rehabilitation therapy. The establishment of China's rehabilitation higher education system and the University of Health and Rehabilitation Sciences as the basis for promoting the development of a rehabilitation therapy team can well promote the aging response and meet the rehabilitation therapy needs of the population.

### Policy emphasis has become an important means to promote rehabilitation education

In recent years, China has issued a series of policies related to the health and pension system of the elderly, which has played a major role in promoting the development of China's health field, especially rehabilitation. In 2013, the introduction of policy documents such as “Several Opinions of the State Council on Accelerating the Development of the Old-age Service Industry” and “Several Opinions of the State Council on Promoting the Development of the Health Service Industry” has rapidly promoted the development of rehabilitation and therapy education in China. From Table 2, it can be seen that after 2013, a large number of rehabilitation therapy-related majors in Chinese colleges and universities emerged. In 2016, the introduction of the “Several Opinions of the State Council on Accelerating the Development of the Rehabilitation Assistive Device Industry” and the subsequent “Notice of the State Council on Printing and Distributing the “13th Five-Year Plan” National Aging Cause development and Pension System Construction Plan” once again promoted the overall rapid development of rehabilitation and therapy education in China.

Currently, public health and healthcare policymakers and leaders should fully carry out rehabilitation medical research, clarify the existing rehabilitation therapy education development problems and weak links, and promote the continuous growth of rehabilitation therapy talents and the continuous improvement of disciplinary capabilities. Increase the training of rehabilitation therapy talents, improve the construction of rehabilitation universities, and increase the training and training of compound rehabilitation talents. At the same time, we will actively promote the construction of rehabilitation medical professional personnel training bases, promote the improvement of policies related to the access standards for rehabilitation therapists, actively set up rehabilitation therapy related majors and related secondary disciplines and interdisciplinary disciplines, and cultivate more high-quality rehabilitation therapy professionals.

### Improving the concept of education is the top priority in the development of rehabilitation education

Public health and healthcare policymakers and leaders attach great importance to the development of physicians' practical ability, emphasizing that educational teaching concepts and training goals should be based on actual needs (21) while adjusting to the latest international rehabilitation concepts and their own disciplinary development advantages. The international community has taken ICF as the basis for the development of rehabilitation science and has built a new rehabilitation education discipline system and rehabilitation education curriculum system according to the classification system of human function and disability. Most of the existing rehabilitation education concepts, goals, and curriculum settings in China refer to the “Rehabilitation Therapy Professional and Technical Personnel Access Standards” and the “Undergraduate Rehabilitation Therapy Professional Education Setting Standards” published in China in 2004 and the examination content of the rehabilitation therapist's professional qualification certification examination, which is lacking in terms of comprehensive competence such as practical operation methods and collaboration with relevant clinical disciplines, and it is difficult to meet the current population rehabilitation therapy needs.

Reference can be made to the WHO International Classification of Functioning, Disability and Health (ICF) (22) theories and methods of function and disability, The characteristics of the development of contemporary rehabilitation disciplines, and the demand for therapists in the field of rehabilitation and the requirements of the WHO Rehabilitation Competency Framework (RCF) (23) for rehabilitation disciplines and rehabilitation services in different practice scenarios. At the same time, the teaching of the basic theories and methods of Traditional Chinese medicine unique to China is added, such as acupuncture and Tuina.

### Improving the comprehensive quality of rehabilitation therapy personnel

The overall training level of rehabilitation therapy talents in China still needs to be improved. With the continuous increase in people's demand for rehabilitation therapy services, the comprehensive quality requirements for rehabilitation therapy personnel are getting higher and higher, and it can be seen from Figure 1 that the number of rehabilitation therapy education masters and doctoral colleges in China is far lower than the professional (vocational) level, and the training direction is not as rich as the professional (vocational) level. This may be related to the late start and short development time of rehabilitation education in China.

We should actively respond to the national policy to cultivate high-level rehabilitation and therapy talents, and cultivate rehabilitation therapy masters and doctoral students in a targeted manner in combination with the national conditions of our country, and gradually improve the academic level of rehabilitation therapy talents. In addition, due to the limitations of undergraduate science degrees in rehabilitation therapy in China, the training direction of its graduates is limited to academic masters, and the training of clinical skills and practical operations is less, which to a certain extent leads to students failing to achieve a better combination of theory and practice. Therefore, relevant colleges and universities should improve the training program for master's degree students in rehabilitation therapy related majors, standardize the design of clinical courses for graduate students, and promote standardized training in related majors. For example, strengthen the supporting construction of laboratories (24), establish professional training rooms such as PT, OT, ST, rehabilitation engineering, traditional rehabilitation of traditional Chinese medicine, etc., actively carry out practical training courses, take the cultivation of students' vocational ability as the core, and evaluate students in various forms such as theoretical examinations, course assessments, internship reports, and internship appraisals, so that the training of students can achieve more ideal results.

### Referring to international rehabilitation standards and developing Chinese characteristics

The degree of standardization of rehabilitation-related professional education in China is not easy to be in line with international standards. The current mature international rehabilitation education model is to cultivate separately according to PT and OT and set up different professional types under them (25). There are only five universities in mainland China that have carried out PT, OT sub-professional classification, and obtained WCPT and WFOT dual international certification, such as Capital Medical University, and most colleges and universities and higher vocational colleges and universities have not yet met the minimum standards of rehabilitation education in the alliance, and even have not yet enrolled students in rehabilitation therapy subtype majors.

With the national “Healthy China 2030” policy orientation and the need for continuous precision in the rehabilitation therapy of the population, the reform of rehabilitation education, and the refinement of the teaching of rehabilitation therapy majors is the general trend. In addition, compared with the professional education certification standards required by WCPT and WFOT, there are still many problems in the current training programs and curriculum systems of China PT and OT that need to be revised and improved. We should keep up with the development trend of international higher education, based on the WCPT and WFOT international curriculum certification standards, carry out the revision of the core curriculum program of PT and OT majors and further optimization of the curriculum system, and at the same time give full play to the unique advantages of rehabilitation therapy in China, add traditional Chinese rehabilitation therapies such as acupuncture and Tuina to the curriculum, and explore the path suitable for rehabilitation therapy education in China on the basis of learning from international experience.

### Adapting the curriculum according to the health needs of the population is the trend of future development

Currently, the curriculum of rehabilitation therapy related specialties in China still has some limitations, such as the insufficient connection between the curriculum content and the necessary professional knowledge points of vocational technology, insufficient depth and breadth of knowledge, the structural lack of talent quality training, and the lack of targeted practice courses. With the support of the state and the efforts of talents from all sides, it is urgent to carry out the development and reform of the curriculum of rehabilitation therapeutics in full consideration of China's national conditions.

With reference to Japan, where aging is very serious, some colleges and universities pay much more attention to elderly rehabilitation than other rehabilitation courses, accounting for more class hours, which indicates that Japanese curriculum content settings have a precise understanding of social applicability. Taking Japan International Welfare University as an example, it has set up teaching and practical courses in different stages for gerontology, geriatrics, geriatric physical therapy, occupational therapy for the elderly, occupational therapy for the elderly, and other disciplines (26). In the future, the curriculum structure of rehabilitation therapy-related specialties in China should fully take into account the social background of the accelerating aging and high incidence of chronic diseases, and build a comprehensive curriculum system based on the health needs of the population, market employment, and knowledge, skills and quality that meet the requirements of job standards. In terms of curriculum content, it is also necessary to fully consider the development characteristics of rehabilitation therapy in China and the national health needs, and emphasize the combination of traditional Chinese medicine and western medicine, as well as the combination of institutional rehabilitation and community rehabilitation.

### Continuous and orderly development of standardized training after graduation and continuing education in practice has become the consensus in the industry

Currently, most medical colleges and universities in China have not jointly carried out standardized training for rehabilitation therapists after graduation with their affiliated hospitals, which is not conducive to the improvement of the individual professional abilities of rehabilitation therapists and the progress of rehabilitation therapy. In 2017, the expert group of the Rehabilitation therapy Professional Committee of the Chinese Rehabilitation Medical Association put forward the expert consensus on the standardization of Chinese rehabilitation therapists and believed that the standardized training of rehabilitation therapists was imperative. Standardized training after graduation should achieve the following points: First of all, it is necessary to define the training objectives. Second, we should formulate a sound system of teacher selection and qualification, teacher training and appointment, and teacher evaluation. Third, standardize the training time and management system of students. Finally, a post-competence examination shall be arranged after the training, and those who pass the examination shall be issued with corresponding post-competence certificates.

As for the continuing education of rehabilitation therapists, the current education methods of short-term training and regular refresher education have been developed more maturely in China. The content of the existing training and refresher learning includes theoretical and technical explanations and case discussions of various types of rehabilitation therapy, greatly improving the theoretical and practical level of on-the-job rehabilitation therapists. However, due to such factors as funds and platforms, the popularity of short-term training and regular refresher education in grass-roots hospitals, community rehabilitation centers, and other places still needs to be improved. In the future, it can also be developed in the following aspects: First of all, the government should promote the construction of an academic exchange platform for the education and training of rehabilitation therapists including medical institutions at all levels, and promote that rehabilitation therapists in primary medical institutions can also continuously improve their professional knowledge and skills. Second, we should improve the professionalism and pertinence of the content of online training courses for rehabilitation therapists, and encourage rehabilitation therapists to actively participate in online education courses, such as Moo classes. Finally, enrich the training content, for example, regularly hold research and training classes for senior management talents of rehabilitation therapists to improve the quality of management cadres.

This study had a few limitations. First of all, this study only introduces the professional education system related to mainland China rehabilitation therapy and lacks comparison with similar projects in Hong Kong, Macao, and Taiwan. Second, the development of master's and doctoral higher education in China's rehabilitation-related majors has only been 7 years, and experience and lessons coexist. Therefore, this study hopes to incorporate more recommendations and opinions to aid in the development of higher education in rehabilitation therapy.

## Conclusion

Rehabilitation education is essential to improve the overall quality of rehabilitation staff, which in turn directly affects the satisfaction of many health needs brought about by aging and chronic diseases, disabilities, and postoperative dysfunction. From the perspective of China's rehabilitation education system, this study uses daily data from Chinese and international rehabilitation therapy-related professional institutions to study the current development status of rehabilitation therapy education in China and summarizes the rehabilitation therapy education model with Chinese characteristics that are suitable for the development of rehabilitation therapy in China in comparison with international rehabilitation education. On the one hand, based on ICF, RCF, and other international rehabilitation-related classification concepts and method guidance, it can promote the goals and system settings of rehabilitation therapy education and training in China to continuously meet the current health needs of the population. On the other hand, based on national conditions, the basic theories and methods of traditional Chinese medicine with Chinese characteristics should be integrated into the training of disciplines related to rehabilitation and therapy. It is also necessary to accelerate the promotion of rehabilitation education in national policies, and at the same time further optimize the existing rehabilitation discipline majors and curriculum settings in accordance with the requirements of relevant international education majors and curriculum settings, and enhance the international recognition of Rehabilitation Therapy education training and personnel qualifications in China. Most of the previous research has focused on a certain level of rehabilitation education, and this article innovatively summarizes the professional education related to rehabilitation in China from the aspects of the full-time (vocational)–undergraduate–master–Ph.D. high-level education system, providing a better reference for health policymakers, leaders, and scholars to formulate a more realistic rehabilitation therapy education Chinese program, and even the formulation of internationally recognized rehabilitation therapy education projects.

## Data availability statement

The original contributions presented in the study are included in the article/supplementary material, further inquiries can be directed to the corresponding author/s.

## Author contributions

SS conducted the study design, analysis, and basic writing. XL collected the data. XO conducted the analysis. WC, PG, ZL, LJ, QG, and ZD gave advice on the statistical analysis and data processing and offered comments to modify the manuscript. QJ and JZ contributed to the study design and supervised the process. All authors read and approved the final manuscript before submission.
